# Explaining changes in wealth inequalities in child health: The case of stunting and wasting in Nigeria

**DOI:** 10.1371/journal.pone.0238191

**Published:** 2020-09-14

**Authors:** Chijioke O. Nwosu, John Ele-Ojo Ataguba

**Affiliations:** 1 The Impact Centre, Human Sciences Research Council, Cape Town, Western Cape, South Africa; 2 Health Economics Unit, School of Public Health and Family Medicine, University of Cape Town, Cape Town, Western Cape, South Africa; Western Sydney University, AUSTRALIA

## Abstract

**Background:**

Malnutrition is a major cause of child death, and many children suffer from acute and chronic malnutrition. Nigeria has the second-highest burden of stunting globally and a higher-than-average child wasting prevalence. Moreover, there is substantial spatial variation in the prevalence of stunting and wasting in Nigeria. This paper assessed the socioeconomic inequalities and determinants of the change in socioeconomic inequalities in child stunting and wasting in Nigeria between 2013 and 2018.

**Methods:**

Data came from the 2013 and 2018 Nigeria Demographic and Health Survey. Socioeconomic inequalities in stunting and wasting were measured using the concentration curve and Erreygers’ corrected concentration index. A pro-poor concentration index is negative, meaning that the poor bear a disproportionately higher burden of stunting or wasting than the wealthy. A positive or pro-rich index is the opposite. Standard methodologies were applied to decompose the concentration index (C) while the Oaxaca-Blinder approach was used to decompose changes in the concentration indices (ΔC).

**Findings:**

The socioeconomic inequalities in child stunting and wasting were pro-poor in 2013 and 2018. The concentration indices for stunting reduced from -0.298 (2013) to -0.330 (2018) (ΔC = -0.032). However, the concentration indices for wasting increased from -0.066 to -0.048 (ΔC = 0.018). The changes in the socioeconomic inequalities in stunting and wasting varied by geopolitical zones. Significant determinants of these changes for both stunting and wasting were changes in inequalities in wealth, maternal education and religion. Under-five dependency, access to improved toilet facilities and geopolitical zone significantly explained changes in only stunting inequality, while access to improved water facilities only significantly determined the change in inequality in wasting.

**Conclusion:**

Addressing the socio-economic, spatial and demographic determinants of the changes in the socioeconomic inequalities in child stunting and wasting, especially wealth, maternal education and access to sanitation is critical for improving child stunting and wasting in Nigeria.

## Introduction

Child malnutrition is a major cause of morbidity and mortality, with nutrition-related factors responsible for about 45% of all under-five deaths globally [1]. In 2018, about 149 million under-five children were stunted, almost 50 million wasted, while over 340 million suffered from hidden hunger (i.e. deficiencies in essential micronutrients) [2]. It is not surprising that one in three children under the age of five years does not grow optimally due to malnutrition [2]. Wasting, resulting from a rapid weight loss or failure to gain weight, confers a weakened immune system and susceptibility to long-term developmental delays as well as an increased risk of mortality on children. Stunting is a long-term nutritional problem that may result in irreversible adverse developmental outcomes and a diminished future earning potential [3].

The probability of nutritional deficiency is higher among children from poor homes, which may lead to a serious risk of an intergenerational perpetuation of the vicious cycle of poverty and undernutrition [4]. Tackling nutritional deficiencies, therefore, has obvious economic returns. For instance, every dollar spent in reducing stunting in high burden countries yields the equivalent of US$18 in economic return [2]. Thus, the challenge posed by nutritional deficiencies, especially stunting and wasting, goes beyond the health system as it exerts a significant toll on individuals, households and the economy. Addressing nutritional deficiencies, therefore, can result in significant socioeconomic benefits.

Nigeria has a significant burden of child malnutrition. Data from the 2018 National Nutrition and Health Survey show a national under-five stunting prevalence of 32%, the second-highest globally [5]. Also, Nigeria has one of the highest child wasting prevalence rates in Africa, estimated at 10.8% in 2016–2017 [6]. Similarly, the underweight prevalence among children, estimated at 19.9% in 2018, was higher than the global average of 15% [5]. Unfortunately, there has been little or no progress in improving these indicators over time. For instance, the prevalence rates for stunting have virtually stagnated at about 30% since 2014, while the prevalence of underweight in children showed no change since 2015.

Child malnutrition varies widely within countries and usually follows the fault line of economic deprivation [5]. In Nigeria, while the national prevalence of stunting was estimated at 32% in 2018, some northern states have prevalence rates that exceed the WHO’s critical value of 40% [5]. This contrasts with an average of 17% prevalence rate in the southeastern states of Nigeria [5]. As Nigeria aims to achieve the health-related Sustainable Development Goals (SDGs), it is important to assess the progressive realisation of these goals. Critically, such an assessment must demonstrate how the gaps in child health outcomes between different groups including the poor and rich, have changed over time.

Some studies have analysed the socioeconomic inequalities in various health outcomes, including stunting and antenatal care services in Nigeria [7, 8]. However, there is a paucity of recent evidence on the critical aspect needed to monitor the progressive realisation of the goal to improve child health outcomes. Changes in the socioeconomic inequalities in child health outcomes over time as well as the key determinants of such changes remain critical for policy to improve child health outcomes in Nigeria. Except for a paper that used dated data to assess socioeconomic inequalities in child health in Nigeria between 1990 and 2008 [9], research in this area is scanty. In addition to the dated data, that paper [9] only used child health indicators like vaccination, diarrhoea and fever/cough treatment, and the changes in the socioeconomic inequalities in the child health indicators were not decomposed to explain factors that underlie such temporal changes. The authors have acknowledged these as limitations in their paper and they form the point of departure for this paper that focuses on stunting and wasting. To our knowledge, this is the first study to assess the factors that underlie temporal changes in the socioeconomic inequalities in key child health outcomes like stunting and wasting in Nigeria.

## Materials and methods

### Data and key variables

Data come from the 2013 and 2018 rounds of the nationally representative Nigeria Demographic and Health Survey (NDHS), the two most recent rounds of the NDHS. A stratified three-stage cluster design was used for the 2013 NDHS, comprising 904 primary sampling units (PSUs) which served as the clusters: 372 urban and 532 rural. The 2013 sample selected 40,680 households, where a minimum of 943 interviews were completed in each state. (Nigeria has 36 states and an autonomous Federal Capital Territory). A more detailed account of the dataset is also available elsewhere [10]. The 2018 NDHS was conducted between August and December 2018 via a two-stage stratified cluster sampling design, with each cluster or PSU defined based on enumeration areas from the 2006 population census frame. The survey, which was billed to be conducted in 1 400 clusters comprising 580 urban and 820 rural clusters, actually took place in 1 389 clusters due to feasibility issues. In total, 41 821 (13 311) eligible women (men) were interviewed as part of the 2018 NDHS. A more detailed description of the 2018 NDHS sampling design is available elsewhere [11]. Both datasets allow for the consistent measurement of indicators at the national, zonal (i.e. the six geopolitical zones) and state levels, while survey weights were appropriately calculated to ensure national representativity.

This paper used the Birth Recode data file, which contains information on every child ever born to each interviewed woman (up to a maximum of 20 children). This data file also links children to their mothers, and it contains other relevant household information.

For each NDHS round, the analysis was restricted to the sample of children aged 0–59 months. After data cleaning, the final sample sizes were 23,992 and 11 150 children in the 2013 and 2018 NDHS, respectively.

The outcome variables are indicators of child stunting and wasting. Both follow their respective standard definitions. A child with a height-for-age *z*-score that is less than the negative of twice the standard deviation of the WHO Child Growth Standards median is considered stunted. A child is wasted if the weight-for-height *z*-score is less than the negative of twice the standard deviation of the WHO Child Growth Standards median [12].

Given that the NDHS does not contain household expenditure information, this paper used the wealth index created in the DHS dataset as an indicator of a child’s socioeconomic status [10]. The wealth index in the NDHS was obtained by applying principal components analysis on household assets [13], accounting for rural-urban differences.

Variables used to decompose the change in stunting and wasting were the child’s characteristics (age, sex and religion), mother’s characteristics (education, marital and employment status), and household characteristics (wealth, location, sanitary condition, dependency ratio, and the characteristics of the household head). These variables are correlated with child nutritional status in prior empirical studies. For instance, a child’s age is associated with a child’s nutritional health outcome [14, 15]. Similarly, a child’s sex is associated with nutritional health outcomes, particularly stunting [16–18], while religion and culture are important correlates of a child’s nutritional outcomes [19, 20]. Furthermore, a child with an educated mother is less likely to be stunted or wasted [21–23]. Other mothers’ characteristics like marital status and employment are also associated with children’s nutritional status [24, 25].

Children’s nutritional health outcomes are also affected by household characteristics like wealth and sanitary conditions [15, 21, 23]. In this paper, sanitary conditions were proxied by a household’s water and toilet conditions. Improved water and toilet conditions were created based on the WHO/UNICEF Joint Monitoring Programme for Water Supply, Sanitation and Hygiene classification [26]. A child’s location is also associated with the child’s health status [27–29]. Moreover, the age and sex of the household head are correlated with children’s nutritional outcomes [30, 31], while high dependency ratios are detrimental to children’s health outcomes [32].

### Analytical methods

#### Descriptive statistics

Basic descriptive statistics were computed and compared between 2013 and 2018. Percentages were computed for categorical variables while mean values were reported for continuous/count variables. Even though there may be skewness, the mean of continuous/count variables were reported to ease comparison with results in the literature.

#### Concentration curves

A concentration curve, suited to assess socioeconomic inequalities [33], depicts the cumulative shares of stunted or wasted children against the cumulative population shares, ranked by socioeconomic status. A line of equality is depicted by a 45-degree line, with a proportional concentration curve coinciding with this line. A pro-rich concentration curve—which depicts a disproportionate concentration of stunting or wasting on the rich—lies below the line of equality. Conversely, a pro-poor curve indicating the disproportionate concentration of stunting or wasting on the poor lies above the line of equality [34].

#### Concentration indices

The concentration index, obtained from the concentration curve, is twice the covariance between the health outcome and the fractional rank in the wealth distribution, divided by the mean of the health outcome indicator [34–37]:

$$C_{H}=\frac{2}{\mu_{H}}cov(H,r)$$ (1)

where *C*_*H*_ refers to the concentration index of the health outcome (*H*); *μ*_*H*_ refers to the mean of the health outcome (i.e. stunting or wasting), and *r* is the fractional rank of the individual/household in the wealth distribution. For continuous outcomes, the index lies in the [-1, +1] interval. A negative (positive) index typically indicates a pro-poor (pro-rich) distribution of stunted or wasted children, while a zero index indicates perfect equality. A zero concentration index may also result from a complex relationship where the concentration curve crosses the line of equality [34].

For categorical variables like indicators of stunting and wasting, the concentration index should be normalised as it may not lie between -1 and +1 [38]. The Erreygers’ normalisation approach was used in this paper [39, 40] to obtain the Erreygers’ normalised concentration index (*E*_*C*_) as follows [41]:

$$E_{C}=4(\frac{\mu_{H}}{b-a})C_{H}$$ (2)

where *a* and *b* refer to the lower and upper limits of the ordinal health indicator, respectively; and *μ*_*H*_ and *C*_*H*_ remain as earlier defined.

Changes in the concentration indices or curves between 2013 and 2018 were assessed using the general characterisation of a pro-poor or a pro-rich change or shift laid out by Ataguba [42]. Let *C*_*t*−1_ and *C*_*t*_ represent the value of the concentration indices at time *t* − 1 and *t*, respectively. A pro-poor change in the concentration indices occurs when *C*_*t*−1_ > *C*_*t*_ while a pro-rich change corresponds to *C*_*t*−1_ < *C*_*t*_.

Only two indices are suitable for assessing relative socioeconomic inequalities in health—the slope index of inequality and the concentration index [33]. They are consistent with ranking individuals across socio-economic groupings, sensitive to changes in population distribution across socio-economic groups and consistent in the distribution of stunting and wasting across the distribution of socioeconomic status [33, 43]. The concentration index was used in this paper because it is decomposable to ascertain the factors that significantly explain socioeconomic inequalities in stunting and wasting, which is relevant to policymakers for evidence-based policy interventions.

#### Decomposing socioeconomic inequalities

Socioeconomic inequalities in stunting and wasting were decomposed using the Wagstaff et al.’s [44] approach. The relationship between a child’s health outcomes (i.e. stunting or wasting) (*H*) and associated determinants (*z*) can be denoted as:

$$H_{i}=\varphi +\sum_{k}\beta_{k}z_{ki}+\varepsilon_{i}$$ (3)

where *φ* and *β* are parameters, and *ε* is the error term. Eq (3) was estimated using linear probability models because binary indicators of stunting and wasting were used, with each model appropriately weighted to the population while correcting for heteroscedasticity. For each year, the concentration index in Eq (1) can be decomposed as (the time subscripts are suppressed here to avoid notational clutter):

$$C_{H}=\sum_{k=1}^{K}(\frac{\beta_{k}{\overset{-}{z}}_{k}}{\mu_{H}})C_{k}+(\frac{GC_{\varepsilon }}{\mu_{H}})$$ (4)

where $\left(\frac{\beta_{k}{\overset{-}{z}}_{k}}{\mu_{H}}=\eta_{k}\right)$ denotes the elasticity of stunting or wasting to marginal changes in the *k*-th explanatory variable, while *C*_*k*_ refers to the concentration index of the *k*-th explanatory variable. *GC*_*ε*_ refers to the generalised concentration index of the error term, and $\left(\frac{GC_{\varepsilon }}{\mu_{H}}\right)$ captures the unexplained/residual component.

#### Oaxaca-Blinder decomposition of changes in socioeconomic inequality

While Eq (4) enables us to decompose observed socioeconomic inequalities in stunting and wasting at each point in time, it does not explain temporal changes in such inequalities. Sequel to decomposing the health concentration index for the relevant health outcome in each year as shown in Eq (4), the change in the concentration index can be decomposed between both periods using the Oaxaca-Blinder decomposition as follows [34]:

$$\Delta C=\sum_{k}\eta_{k,t}(C_{k,t}-C_{k,t-1})+\sum_{k}C_{k,t-1}(\eta_{k,t}-\eta_{k,t-1})+\Delta (\frac{{GC}_{\varepsilon ,t}}{\mu_{t}})$$ (5)

where *t* indicates the time period (with the convention that *t* − 1 and *t* denote the first (i.e. 2013) and second (i.e. 2018) periods respectively, while other terms in Eq (5) are as earlier defined).

Eq (5) decomposes the temporal change in the socioeconomic inequality in stunting or wasting into a part that captures the temporal changes in socioeconomic inequality in the determinants of stunting or wasting: ∑_*k*_ *η*_*k*,*t*_(*C*_*k*,*t*_ − *C*_*k*,*t*−1_), and another component that captures the changes in the elasticities of stunting or wasting with respect to these determinants: ∑_*k*_ *C*_*k*,*t*−1_(*η*_*k*,*t*_ − *η*_*k*,*t*−1_). The final term in Eq (5), $\Delta (\frac{{GC}_{\varepsilon ,t}}{\mu_{t}})$, measures the temporal difference in the error/residual component [34]. Note that in Eq (5), the temporal changes in the concentration indices of the predictors/determinants are weighted by the elasticities of the second period, while the first-period concentration indices are used to weight the change in the elasticities. The Oaxaca-Blinder decomposition is not unique [34], as an alternative decomposition would be to weight the difference in the concentration indices by the first-period elasticities and the difference in the elasticities by the second-period concentration indices. Given that there are no analytical standard errors for the estimates derived from Eqs (4) and (5), bootstrap routines [45, 46] with 1,000 replications were used, accounting for the full sampling structure of the NDHS. All analyses were implemented using Stata^®^ [47]. The *conindex* routine was employed in obtaining the Erreygers’ normalised or corrected concentration indices, while accounting for the survey design (clustering, stratification and the resulting survey weights) in each NDHS round [48].

## Results

As shown in Table 1, the prevalence of stunting remained virtually unchanged in both 2013 and 2018 at approximately 36.7%. However, the prevalence of wasting declined by 11.5 percentage points from 18.4% in 2013 to 6.9% in 2018. The average age of children was 28–29 months. There was an almost even split between boys and girls in both rounds while the proportion of working women remained almost unchanged at about 70% in both rounds. About 10% of children lived in female-headed households while almost all their mothers were either married or cohabiting. The proportion of households with access to an improved water source increased from 63% (2013) to 73% (2018) while those with an improved toilet facility increased from 51% (2013) to 56% (2018). The population was predominantly rural, with urban residents comprising 37% and 44% of the population in 2013 and 2018, respectively. There was also widespread, albeit declining maternal illiteracy, with the mothers of 46% and 39% of the children having no education in 2013 and 2018, respectively. Only about 6% and 10% of mothers had post-secondary education in 2013 and 2018, respectively. Islam was the predominant religion, accounting for 60% and 56% of the population in 2013 and 2018, respectively. The North-west geopolitical zone accounted for the highest proportion of the population in both rounds, while the South-east and South-south had the lowest proportions in 2013 and 2018, respectively.

**Table 1:** Descriptive statistics.

Variable	Mean or Percentage		Change in mean/percentage (2018–2013)
	2013	2018	
Stunted	36.7%	36.7%	-0.1
Wasted	18.4%	6.9%	-11.5***
Child’s age (months)	28.7	28.3	-0.4
Household head’s age (years)	40.9	41.2	0.3*
Number of under-5 children in household	2.3	2.2	-0.1***
Male	49.8%	51.0%	1.3*
Mother currently works	70.2%	69.8%	-0.4
Household is headed by a female	9.7%	10.5%	0.9**
Mother’s marital status (= 1 if married/cohabiting; 0 otherwise)	96.2%	95.8%	-0.4
Household has improved drinking water	62.8%	72.7%	9.9***
Household has improved toilet facility	50.9%	55.5%	4.6***
Resides in an urban area	37.0%	44.0%	7.0***
Poorest income quintile	21.9%	18.4%	-3.5***
Poorer income quintile	22.0%	19.6%	-2.4***
Middle income quintile	19.1%	20.9%	1.8***
Richer income quintile	18.8%	21.1%	2.3***
Richest income quintile	18.2%	20.0%	1.8***
Mother has no education	46.4%	38.9%	-7.5***
Mother has only primary education	20.2%	16.1%	-4.1***
Mother has only secondary education	27.4%	35.1%	7.7***
Mother has tertiary education	6.1%	9.9%	3.8***
Religion: Islam	60.3%	56.0%	-4.2***
Religion: Christian	38.8%	43.4%	4.6***
Religion: Traditional/other	1.0%	0.6%	-0.4***
Geopolitical zone: North-Central	14.4%	14.2%	-0.1
Geopolitical zone: North-East	16.8%	15.4%	-1.4***
Geopolitical zone: North-West	34.8%	29.4%	-5.3***
Geopolitical zone: South-East	9.3%	12.7%	3.5***
Geopolitical zone: South-South	9.6%	10.4%	0.8**
Geopolitical zone: South-West	15.3%	17.8%	2.5***
**Sample size**	**23 992**	**11 150**^†^	

Estimates weighted by sampling weights;

^**†**^ The wasting sample size in 2018 was 11 102;

*, **, and *** indicate statistical significance at the 10%, 5%, and 1%, respectively (the statistical significance of the changes (2018–2013) are based on bootstrapped standard errors with 1 000 replications). The mean values have been reported because the median values are not statistically different from the mean values. But other measures of central tendency are available from the authors if needed.

**Fig 1:**
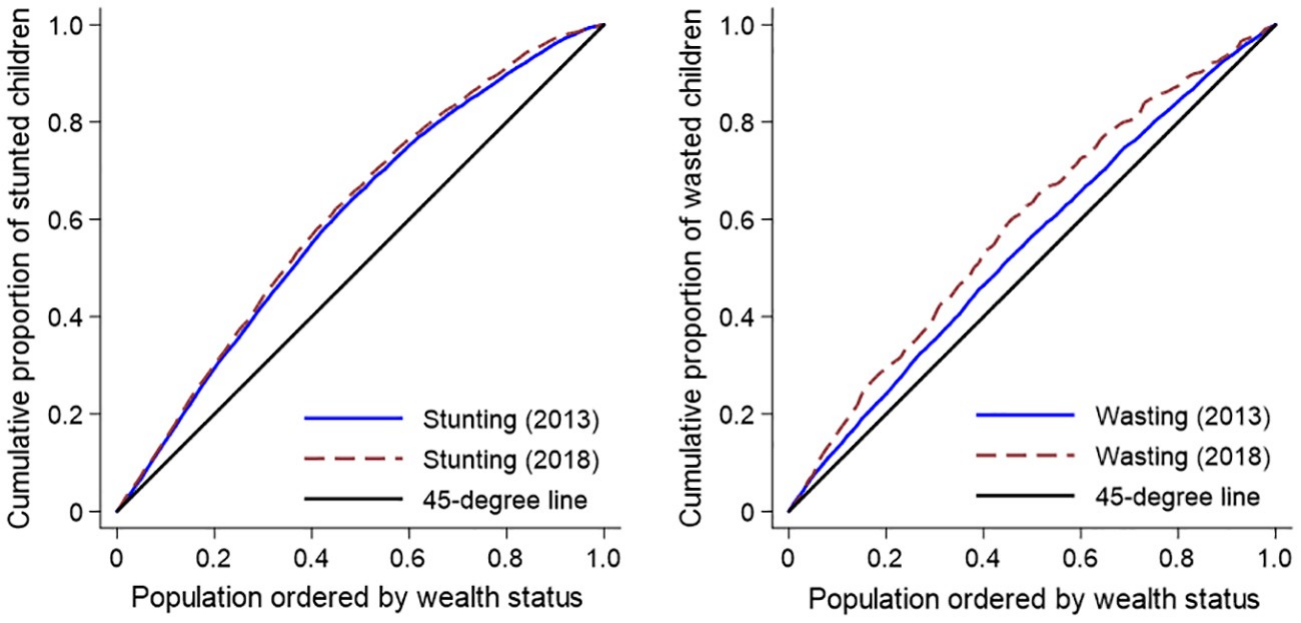
Concentration curves for stunting and wasting, Nigeria, 2013 & 2018. The concentration curves are not adjusted for Erreygers’ correction.

The unadjusted concentration curves in Fig 1 indicate that stunting and wasting were pro-poor in both 2013 and 2018. In other words, both stunting and wasting were disproportionally concentrated on poorer children as the concentration curves lay everywhere above the line of equality. While the stunting curves indicate little or no change in inequality, the wasting curves suggest that wasting became more pro-poor (i.e. more concentrated on the poor) between 2013 and 2018. Stunting and wasting were also more prevalent in the northern geopolitical zones (Table 2). Moreover, both the changes and ratios of the concentration indices across geopolitical zones (Table 2) indicate that the prevalence of stunting generally worsened between 2013 and 2018 while wasting declined.

**Table 2:** Stunting and wasting prevalence rates across geopolitical zones, Nigeria, 2013 & 2018.

	Geopolitical zone	Prevalence rate (%)		Change (2018–2013)	Ratio (2018/2013)
		2013	2018		
**Stunting**	North-central	28.7	27.8	-0.9	1.00
	North-east	42.2	48.6	6.3***	1.15
	North-west	54.8	56.9	2.1*	1.04
	South-east	15.2	18.2	3.0**	1.20
	South-south	18.0	19.9	1.8	1.11
	South-west	21.8	23.0	1.1	1.06
	**National**	**36.7**	**36.7**	**-0.1**	**1.00**
**Wasting**	North-central	12.5	5.7	-6.8***	0.46
	North-east	19.8	9.9	-9.9***	0.50
	North-west	27.3	8.8	-18.4***	0.32
	South-east	12.1	4.7	-7.4***	0.39
	South-south	11.5	4.4	-7.1***	0.38
	South-west	10.2	5.1	-5.1***	0.50
	**National**^†^	**18.4**	**6.9**	-11.5***	**0.38**

Total observations in the sample: 23 992 (2013), 11 150 (2018);

^**†**^ The wasting sample size in 2018 was 11 102;

*, **, and *** indicate statistical significance at the 10%, 5%, and 1%, respectively (the statistical significance of the changes (2018–2013) are based on bootstrapped standard errors with 1 000 replications).

The (Erreygers’) normalised concentration indices depicted in Table 3 do not exactly mirror the results shown in Fig 1. While the national concentration indices for both stunting and wasting indicate pro-poor distributions given the negative concentration indices (in conformity with the concentration curves), the stunting concentration index in 2018 was more pro-poor than that of 2013, resulting in a significant pro-poor change over time. Conversely, the wasting concentration index in 2018 was less pro-poor than that of 2013, resulting in a significant pro-rich change. Thus, the extent to which wasting was disproportionally borne by the poor actually declined between 2013 and 2018 when the qualitative nature of wasting is taken into account, a result masked by the non-adjusted concentration curves in Fig 1.

**Table 3:** Normalized concentration indices (national and zone-specific) for stunting and wasting in Nigeria, 2013–2018.

		2013	2018	Change (2018–2013)
**Stunting**	North-Central	-0.123*** (0.027)	-0.181*** (0.031)	-0.059* (0.032)
	North-East	-0.154*** (0.030)	-0.161*** (0.037)	-0.007 (0.037)
	North-West	-0.134*** (0.021)	-0.182*** (0.025)	-0.048* (0.027)
	South-East	-0.040 (0.028)	-0.144*** (0.027)	-0.104*** (0.033)
	South-South	-0.171*** (0.020)	-0.155*** (0.029)	0.016 (0.032)
	South-West	-0.150*** (0.023)	-0.197*** (0.024)	-0.047 (0.032)
	**National**	-0.298*** (0.013)	-0.330*** (0.014)	-0.032** (0.014)
**Wasting**	North-Central	-0.016 (0.017)	-0.018 (0.012)	-0.002 (0.019)
	North-East	-0.093*** (0.025)	-0.031* (0.017)	0.062** (0.025)
	North-West	0.111*** (0.024)	-0.061*** (0.013)	-0.172*** (0.019)
	South-East	0.003 (0.018)	0.002 (0.014)	-0.001 (0.026)
	South-South	-0.047** (0.019)	-0.025 (0.015)	0.023 (0.022)
	South-West	-0.010 (0.018)	-0.006 (0.013)	0.004 (0.019)
	**National**^†^	-0.066*** (0.010)	-0.048*** (0.007)	0.018* (0.010)

Estimation samples were 23 992 and 11 150 in 2013 and 2018 respectively;

^†^ The wasting sample size in 2018 was 11 102; bootstrapped standard errors with 1000 replications in parentheses (for differences in concentration indices) *, **, and *** indicate statistical significance at the 10%, 5%, and 1%, respectively.

Table 3 also indicates that the stunting concentration indices did not vary widely across geopolitical zones in each year (except for the 2013 index for the South-east). Apart from the South-east, the concentration indices in 2013 ranged from -0.123 (North-central) to -0.171 (South-south). On the other hand, the 2018 stunting concentration indices ranged from -0.144 (South-east) to -0.197 (South-west). The changes in the concentration indices for stunting show that a statistically significant pro-poor change occurred in the North-central, North-west and South-east zones, while the other zones did not experience a statistically significant change in socioeconomic inequality.

For wasting, there was a general concentration of wasting on poorer children in the southern and northern zones with most of the statistically significant concentration indices pro-poor. It was only in the North-west zone that the concentration index switched from a statistically significant pro-rich to a statistically significant pro-poor index between 2013 and 2018, resulting in a significant pro-poor change. On the contrary, the North-east experienced a significant pro-rich change in inequality due to a less pro-poor index in 2018 relative to 2013.

Table 4 reports the contributions of the various components (weighted change in the predictors’ socioeconomic inequalities and weighted change in the elasticities). For brevity, the focus will be on only the first term. (The resulting change in the residuals are available on request). Thus, we are interested in the effect of the changes in the socioeconomic inequalities in the predictors on changes in the socioeconomic inequalities in stunting and wasting. In other words, we will focus on columns I, II, V and VI in Table 4.

**Table 4:** Oaxaca-Blinder decomposition of the change in socioeconomic inequalities in stunting and wasting, Nigeria, 2013–2018.

	Stunting				Wasting			
	I	II	III	IV	V	VI	VII	VIII
	*η*_k,t_Δ*C*_*k*_	%	*C*_k,t−1_Δ*η*_*k*_	%	*η*_k,t_Δ*C*_*k*_	%	*C*_k,t−1_Δ*η*_*k*_	%
Child’s age	0.001 (0.001)	-1.8	>-0.001 (<0.001)	0.1	-0.002 (0.003)	-9.5	>-0.001 (0.001)	-1.4
Household head’s age	>-0.001 (<0.001)	1.2	>-0.001 (<0.001)	0.6	>-0.001 (0.001)	-1.8	0.001 (0.001)	3.1
Number of u-5 in household	0.001** (0.001)	-4.4	-0.003* (0.002)	10.5	<0.001 (0.002)	1.5	-0.002 (0.005)	-9.1
Poorer quintile	0.001* (<0.001)	-2.3	-0.001 (0.003)	2.0	0.003* (0.001)	15.7	0.020** (0.010)	114.1
Middle quintile	0.004*** (0.001)	-12.6	-0.001 (0.001)	2.2	0.007** (0.003)	37.1	-0.004** (0.002)	-24.4
Richer quintile	0.001 (0.001)	-2.0	-0.007 (0.005)	21.1	0.001 (0.001)	5.3	-0.040** (0.016)	-227.2
Richest quintile	-0.005*** (0.002)	15.2	-0.021** (0.010)	66.1	-0.006** (0.003)	-34.6	-0.074*** (0.027)	-417.9
Male	>-0.001 (0.001)	0.4	<0.001 (<0.001)	-0.9	>-0.001 (0.003)	-1.5	0.002 (0.001)	9.6
Primary education	0.001* (0.001)	-4.4	>-0.001 (<0.001)	0.4	0.005** (0.002)	27.5	-0.002 (0.001)	-10.2
Secondary education	0.003** (0.001)	-10.0	-0.020** (0.009)	61.6	0.004 (0.003)	21.1	-0.021 (0.027)	-118.3
Higher education	-0.003*** (0.001)	10.1	-0.004*** (0.001)	13.0	-0.002 (0.002)	-10.5	>-0.001 (0.004)	-2.1
Mother works	>-0.001 (<0.001)	0.2	0.006* (0.003)	-18.1	>-0.001 (0.001)	-0.5	0.004 (0.010)	24.9
Female head	<0.001 (<0.001)	-0.1	<0.001 (<0.001)	-0.5	0.001 (0.001)	4.5	-0.001 (0.001)	-6.9
Mother is married/cohabiting	0.001 (0.001)	-2.1	-0.001 (0.001)	2.4	-0.001 (0.003)	-6.2	0.001 (0.003)	3.4
Christian	0.006*** (0.002)	-18.2	-0.019** (0.009)	59.4	0.004 (0.003)	19.8	0.004 (0.024)	21.4
Traditional/other	>-0.001 (<0.001)	0.0	>-0.001 (<0.001)	0.1	>-0.001* (<0.001)	-0.2	>-0.001 (<0.001)	0.0
Improved water facility	0.002 (0.001)	-6.5	-0.015 (0.017)	48.1	-0.007* (0.004)	-39.3	0.093** (0.045)	520.7
Improved toilet facility	0.006* (0.003)	-18.9	0.030** (0.012)	-94.3	0.001 (0.011)	5.1	-0.031 (0.040)	-173.0
Urban	<0.001 (0.001)	-1.1	0.004 (0.012)	-14.0	0.001 (0.002)	3.7	-0.061* (0.033)	-340.0
Geopolitical zone	0.015*** (0.004)	-45.8	0.017 (0.014)	-53.9	0.002 (0.005)	8.8	0.139*** (0.036)	780.6
**Overall change in CI (ΔC)**	-0.032** (0.014)	**100.0**			0.018* (0.010)	**100.0**		

Δ*C*_*k*_ = *C*_*k*,*t*_ − *C*_*k*,*t*−1_; Δ*η*_*k*_ = *η*_*k*,*t*_ − *η*_*k*,*t*−1_; estimation samples were 23 992 and 11 150 in 2013 and 2018 respectively (the wasting sample size in 2018 was 11 102); survey design accounted for in estimation; standard errors in parenthesis; bootstrapped standard errors with 1000 replications;

*, **, and *** indicate statistical significance at the 10%, 5%, and 1%, respectively.

As shown in Table 4, belonging to the richest wealth quintile and mother’s higher education attainment had significant and nontrivial pro-poor effects on the changes in the socioeconomic inequalities in stunting, with wealth having the bigger impact (15%). (Other predictors with pro-poor but numerically trivial and/or statistically insignificant weighted changes were the age of the household head, sex of the child, mother’s employment status, and adherence to traditional/other beliefs (relative to Islam)).

Conversely, the weighted pro-rich change due to under-five dependency in the household, the poorer and middle wealth quintiles (relative to the poorest), primary education and secondary education, Christian affiliation, access to improved toilet facilities and geopolitical zone of residence mitigated the pro-poorness of stunting between both years. Thus, the pro-rich changes in these variables mitigated the extent to which stunting was disproportionally borne by the poor over time. Geopolitical zone of residence accounted for the largest pro-rich effect (46%), followed by access to improved toilet facilities (19%) and Christian religious affiliation (18%). Other variables with similar effect, but with trivial and/or insignificant coefficients were the child’s age, the richer wealth quintile, female headship, mother’s marital status, access to an improved water source and urban residence.

Belonging to the wealthiest quintile and access to improved water sources had statistically significant and nontrivial weighted pro-poor effects on wasting, accounting for 35% and 39% of the change, respectively. Given that wasting had a pro-rich change over time, these variables dampened the degree of pro-richness of the change in wasting between 2013 and 2018 (thereby disfavouring the poor relative to what would have otherwise been the case). Therefore, eliminating such changes would improve the pro-rich change in wasting over time. Other pro-poor determinants, which were numerically trivial and/or statistically insignificant, were the ages of the child and the household head, child’s sex, mother’s higher educational attainment, employment and marital status, and adherence to traditional/other religion. Conversely, belonging to the poorer and middle quintiles and attaining only primary education had weighted pro-rich changes, resulting in their accentuating the pro-rich change in wasting between 2013 and 2018. These variables accounted for 16%, 37%, and 28% of the observed changes in wasting concentration indices, respectively. Predictors with a similar sign but numerically trivial and/or statistically insignificant effects were the number of under-five children in the household, belonging to the richer quintile, mother attaining secondary education, Christianity, access to an improved toilet source, urban residence and geopolitical zone of residence.

Table 5 depicts the survey round-specific concentration indices and elasticities that informed the estimates in Table 4. These results provide more clarity in interpreting the main results in Table 4, as they reveal the source of the signs of the predictors (whether they are due to the elasticities or change in the concentration indices).

**Table 5:** Elasticities and concentration indices of determinants of the socioeconomic inequalities in stunting and wasting, Nigeria, 2013 & 2018.

	Concentration indices		Elasticities			
			Stunting		Wasting	
	*C*_k,t_	*C*_*k*,*t*−1_	*η*_k,t_	*η*_*k*,*t*−1_	*η*_k,t_	*η*_*k*,*t*−1_
Child’s age	0.004	0.001	0.230	0.263	-0.694	-0.479
Household head’s age	-0.001	-0.006	-0.076	-0.113	-0.067	0.033
Number of u-5 children in household	-0.037	-0.057	0.070	0.010	0.013	-0.016
Poorer quintile	-0.342	-0.301	-0.017	-0.020	-0.068	>-0.001
Middle quintile	-0.026	0.053	-0.051	-0.038	-0.084	-0.002
Richer quintile	0.328	0.336	-0.083	-0.063	-0.122	-0.002
Richest quintile	0.640	0.596	-0.110	-0.075	-0.140	-0.015
Male	0.013	0.015	0.077	0.058	0.171	0.057
Primary education	-0.040	0.054	-0.015	-0.013	-0.052	-0.019
Secondary education	0.434	0.474	-0.080	-0.039	-0.094	-0.050
Higher education	0.272	0.187	-0.038	-0.016	-0.022	-0.020
Mother works	0.126	0.130	0.017	-0.027	0.027	-0.007
Female head	0.032	0.070	-0.001	-0.003	-0.021	-0.003
Mother’s marital status	>-0.001	-0.015	0.046	-0.004	-0.075	-0.035
Christian	0.413	0.459	-0.127	-0.085	-0.076	-0.085
Traditional/other	-0.003	-0.013	-0.001	-0.003	-0.003	-0.004
Improved water facility	0.522	0.567	-0.046	-0.019	0.155	-0.008
Improved toilet facility	0.697	0.544	0.039	-0.016	0.006	0.063
Urban	0.663	0.699	-0.010	-0.017	-0.018	0.068
Geopolitical zone	0.346	0.404	-0.249	-0.292	-0.027	-0.370

Survey design accounted for; bootstrapped standard errors (from 1,000 replications) not reported here but available on request.

## Discussion

This paper has shown that stunting and wasting occur more frequently among poorer children in Nigeria. This finding was consistent in both 2013 and 2018. While the concentration of stunting and wasting is higher among poorer children, more of the poorer children are becoming stunted over time in Nigeria relative to their richer counterparts, resulting in a pro-poor change. Conversely, the extent to which poorer children bear the burden of wasting is declining, resulting in a pro-rich change.

Children in the wealthiest quintile and mother’s attainment of higher education contributed to the worsening of the socioeconomic inequality in stunting over the period by increasing the degree of pro-poorness in stunting between 2013 and 2018. As expected, each of these results was due to a negative elasticity (i.e. weight) and (increasing) pro-rich socioeconomic inequality in these variables (see Table 5). Therefore, eliminating pro-rich changes in these variables and/or the elasticity of stunting to changes in these determinants would mitigate the worsening change in the socioeconomic inequality in child stunting in Nigeria. Had these variables become less pro-rich over time, the change in the socioeconomic inequality in stunting would have been less pro-poor than it was. Thus, the increased wealth inequality and a greater concentration of higher education among the relatively wealthy resulted in a higher concentration of stunting on the poor in 2018 relative to 2013. Results from other developing countries corroborate the negative elasticities of these variables with respect to child stunting. For instance, maternal education was an important determinant of lower stunting odds in India [49]. Similarly, the negative correlation between wealth and stunting in particular, and children’s nutritional health outcomes, in general, is well documented. For instance, stunting odds ratios of 3.36 and 8.35 have been recorded for the poorest children relative to the wealthiest in India and Guatemala, respectively [15].

As indicated in Tablswes 4 and 5, the weighted pro-rich change in maternal secondary education, Christianity, and geopolitical zone were due to a negative elasticity to stunting and less pro-richness in 2018 relative to 2013 while that of improved toilet facilities was due to a pro-rich change and a positive elasticity. The negative elasticities corroborate existing evidence of a negative relationship between these determinants and stunting in earlier studies. For instance, the negative elasticity of stunting to the geopolitical zone fixed effect concurs with prior evidence of the elevated probability of child mortality in northern Nigeria [27]. Moreover, the link between child health and religion has been established elsewhere. In Ghana, for instance, a bivariate analysis indicates that children born to mothers practising the Muslim or traditional religion had a significantly higher risk of death compared to their Christian counterparts—though the relationship disappeared in a multiple regression analysis [50]. Brainerd and Menon [19] suggest that a child’s exposure to in-utero fasting during Ramadan may adversely affect the child’s dietary intake and nutritional health. However, a systematic review and meta-analysis showed that while placental weight was significantly lower among Ramadan-fasting mothers, there was no significant difference in birth weight between the children of fasting and non-fasting mothers [51].

In addition to maternal education that may lower stunting odds as reported in India [49], sanitation and hygiene are risk factors that also contribute to stunting differentials between the wealthy and the less wealthy [21]. Moreover, the positive elasticity of stunting to the number of under-five children concurs with prior evidence on the positive relationship between stunting and the number of siblings a child has, thus reflecting the effect of greater competition for available household resources [22]. Generally, the “improvement” in the weighted inequalities in these determinants of stunting between 2013 and 2018 resulted in less pro-poor inequality in stunting than would have been the case.

The negative elasticity of wealth to nutritional health problems reported for stunting also applies to the pro-poor wealth effect on wasting, a finding which is in line with prior evidence from India [15]. However, the pro-poor effect of improved water source was caused by a combination of a positive elasticity of wasting and a decline in the extent of the pro-richness of access to improved water source between 2013 and 2018. Indeed, the positive elasticity of wasting to access to an improved water source is not supported by most of the evidence found in previous studies [21]. However, the declining pro-richness of access to an improved source of water may be due to improvements in access to improved water sources in Nigeria in the recent past [10, 11].

Conversely, the weighted pro-rich effects of the poorer and middle wealth quintiles, as well as mothers only attaining primary education, were due to negative wasting elasticities and pro-poor changes in these predictors’ concentration indices. Thus, compared to the poorest group, children from poorer and middle wealth groups were less likely to be wasted, a finding that concurs with the negative relationship between wealth and wasting found elsewhere [15].

Forty years since the Alma Ata Declaration, the state of child health remains dire in Nigeria, with a state of emergency already declared on maternal and child mortality [52]. The country loses far too many under-five children, making it one of the largest contributors to global under-five mortality rates [52]. Moreover, malnutrition is the underlying cause of many instances of child morbidity and mortality in the country [53]. Malnutrition is not only a health sector problem; as demonstrated above, socioeconomic factors like wealth, education, religion, location, sanitary conditions and child dependency, that are mainly the social determinants of health inequalities [54], play a significant role in children’s nutritional outcomes and inequalities. It is of utmost importance to bridge the gap in child nutrition outcomes between relatively affluent children and their poorer counterparts. Unfortunately, wealth-related inequalities in child nutritional outcomes still significantly disfavour the poor, with a worsening situation for stunting while only decelerating for wasting. The results in this paper and evidence from the literature point to an opportunity to reduce the concentration of child stunting and wasting among poorer children in Nigeria through improving key socioeconomic conditions of mothers, including sanitation, hygiene and access to quality education, especially for mothers from poorer backgrounds. It is important, therefore, to recognise the multisectoral nature of child malnutrition in designing an appropriate response.

### Strengths, limitations and areas for future research

This paper uses comparable datasets collected using the same methodology to compare the patterns in child stunting and wasting between 2013 and 2018. The paper demonstrates how socioeconomic inequalities in stunting and wasting in Nigeria have changed between both periods. Besides, the decomposition analysis allows for an assessment of factors that explain changes in socioeconomic inequalities, an issue of significance in the discourse of progressive realisation of child health as entrenched in the SDGs. Although this is the first study to assess these changes in socioeconomic inequalities in stunting and wasting in Nigeria, it has a few limitations. The DHS dataset that is standardised across countries limits its ability to fully account for the local context when designing questions [55]. As shown in this paper, location is associated with child stunting and wasting, which may require a more nuanced analysis by geopolitical zones in Nigeria. Also, while child health could be assessed using other indicators and monitored over time, this paper only used stunting and wasting as indicators, respectively, for long- and short-term nutritional deficiencies. Thus, this paper suggests other analyses for future research to provide a ‘holistic’ view of inequalities in child health outcomes, and for policy to address the significant burden of child ill-health in Nigeria. The complementary analyses should investigate the changing patterns in other key indicators of child health, including underweight, overweight and obesity. Besides, the intergenerational transfer of overweight and obesity [56] could be assessed in Nigeria.

## Conclusion

This paper has shown that children from poorer backgrounds consistently bore a disproportionately higher burden of stunting and wasting in Nigeria between 2013 and 2018. Although the extent to which wasting occurs more frequently among poorer children in Nigeria declined over the study period, the burden of stunting disproportionately increased among the poor. Importantly, the significant factors that drive the changes in socioeconomic inequalities in stunting and wasting, especially family wealth, maternal education, sanitary conditions and location, lie outside the health sector. A deeper understanding of these social determinants of health inequalities and addressing them will significantly reduce the burden of child stunting and wasting and substantially improve child health outcomes in Nigeria.
